# Scattering of N_2_ Molecules from Silica Surfaces: Effect of Polymorph and Surface Temperature

**DOI:** 10.3390/molecules27217445

**Published:** 2022-11-02

**Authors:** Maria Rutigliano, Fernando Pirani

**Affiliations:** 1 CNR-ISTP (Istituto per la Scienza e Tecnologia dei Plasmi), Via Amendola 122/D, 70126 Bari, Italy; 2 Dipartimento di Chimica, Biologia e Biotecnologie, Università di Perugia, Via Elce di Sotto 8, 06123 Perugia, Italy; 3 Dipartimento di Ingegneria Civile ed Ambientale, Università di Perugia, Via G. Duranti 93, 06125 Perugia, Italy

**Keywords:** molecular dynamics simulations, long-range interactions, potential energy surface, surface processes, roto-vibrational distributions, reaction mechanism

## Abstract

The inelastic scattering of N_2_ molecules from silica surfaces, taken at 100 K, has been investigated by adopting a semiclassical collision model in conjunction with the appropriate treatment of the long-range interaction forces. Such forces promote the formation of the precursor state that controls all basic elementary processes occurring at the gas–surface interphase. The probabilities for the different elementary surface processes triggered by quartz are determined and compared with those recently obtained for another silica polymorph (cristobalite). In addition, the final roto-vibrational distributions of N_2_ molecules undergoing inelastic scattering have been characterized. N_2_ molecules, impinging on both considered surfaces in low-medium vibrational states, preserve the initial vibrational state, while those inelastically scattered are rotationally excited and translationally colder. The surface temperature effect, investigated by raising the temperature itself from 100 K up to 1000 K, emerges more sharply for the cristobalite polymorph, mainly for the molecules impinging in the ground roto-vibrational state and with low collision energies.

## 1. Introduction

The inelastic scattering of molecules from surfaces triggers several energy exchanges that can have repercussions on the energetics and collision dynamics in gaseous and plasma environments. Moreover, this process exhibits some selectivity and propensity strictly related to the alignment of impinging molecules and to the surface roughness seen by the gaseous molecules.

Several studies exist within this context, recently conducted by different groups, to characterize the inelastic scattering of molecules both with increasingly complex and refined experiments [1,2,3,4,5,6] and with state-resolved molecular dynamics (MD) simulations, the latter based on quantum [7,8], quasi-classical trajectory (QCT) [9] and semiclassical [10,11,12,13,14] methods.

Silica is a highly versatile and very common material in natural, laboratory and industrial environments. Traces of SiO_2_ are found in grains of spatial origin [15], but silica-based materials are also of interest for the thermal protection panels of space vehicles [16], micro-nano electronics [17], and nano-medicine [18]. Accordingly, the investigation of the interaction of small molecules, such as H_2_, O_2_, N_2_, CO with this kind of surface, appears mandatory for the advancement of knowledge in the different fields of scientific and technological interest.

In ref. [19], a strong correlation has been found between the incidence speed, angle, surface topology and the probability distribution of accommodation coefficients for the interaction of nitrogen molecules with silica (quartz). There, MD simulations were performed by using the ReaxFF (reactive-force-field) tool in which van der Waals’s interactions are simulated through a distance-corrected Morse potential. Except for this last work, to our knowledge, there are no other works in the literature dealing with the dynamics of the interaction of N_2_ with silica, although some results on the simulation of adsorption exist [20,21,22].

Therefore, this paper aims to shed light on the interaction dynamics of nitrogen molecules on silica surfaces, highlighting the mechanisms underlying the process at the atomic level. Our objective is to understand whether the result of the collision depends on the used polymorph or on the surface temperature (T_S_), rather than on the initial roto-vibrational (j_i_, v_i_) state of the incident molecule. At the same time, it will also be shown how different surface chemical compositions influence the interaction driving the dynamics of impinging species. These objectives will be achieved by introducing an appropriate treatment of the long-range forces playing a primary role in the formation of precursor states. An accurate investigation of nitrogen (atomic and molecular) interactions with different silica surfaces at different temperatures appears mandatory in the research fields for which this system is of interest, such as aerospace, environment and plasma-assisted catalysis. In these fields, roto-vibrational excited molecules, scattered from the surface, can play a leading role in collisions with other species in the gas-phase or plasma Therefore, the assessment of the internal states of molecules, only obtainable with a state-to-state model such as the one used here, is of considerable importance.

These tasks will be carried out through MD simulations with the adoption of a semiclassical collision method [23] in conjunction with an accurate potential energy surface (PES) formulation, obtained by treating the interaction at short and long distances differently. Recently, it has been shown that long/intermediate-range interactions have a considerable impact on the microscopic dynamics of molecules interacting with surfaces [12,13,14]. Moreover, the appropriate treatment of the weak interactions is of primary importance to accurately describe the dynamics of several elementary processes, including those occurring at the gas–surface interphase, especially for molecules impinging at low collision energy [24,25]. The adopted model to properly describe the long-range interaction is the improved Lennard–Jones (ILJ) model, postulated in ref. [26] and already used in refs. [10,11,12,13,14], to study the dynamics of light molecules impinging on graphite. The obtained results are consistent with those of the molecular alignment processes, associated with the propensity and selectivity of elastic and inelastic scattering, observed in gas-phase experiments conducted with molecular beams [2,27,28].

The paper is organized as follows. In Section 2, we focus on the results obtained in determining ILJ potential for N_2_ interacting on the SiO_2_ surface and in the building of PES. Moreover, the results of MD simulations obtained for N_2_ colliding with quartz are presented and discussed by comparing them with those recently obtained for cristobalite [29]. Then, for both polymorphs, we deal with the surface temperature effect, mainly dwelling on the scattering probabilities and rotational distributions of the scattered molecules. In addition, some comments and considerations are drawn. Section 3 provides all basic details of the methods employed to carry out MD simulations, to formulate the PES and to estimate the interaction potential parameters. Finally, Section 4 summarizes our conclusions. 

## 2. Results and Discussion

### 2.1. The Long-Range Potential of N and N_2_ Interacting with Silica

The values of the potential parameters, here exploited to formulate through the ILJ function the asymptotic no-covalent part of the interaction, are reported in Table 1 of ref. [29]. Such values have been evaluated considering some basic physico-chemical properties of N, N_2_ and the SiO_2_ unit, as their electronic polarizability. In the Section 3, details are given on the procedure adopted and on the comparison of determined values with data available in the literature.

The complete V_N2-silica_ potential is obtained by adding a Coulomb term to the interaction component, represented by the ILJ function, accounting for the electrostatic contribution, due to the quadrupole moment of the nitrogen molecule interacting with the anisotropic charge distribution of the surface, according to what has been performed in ref. [20].

**Figure 1:**
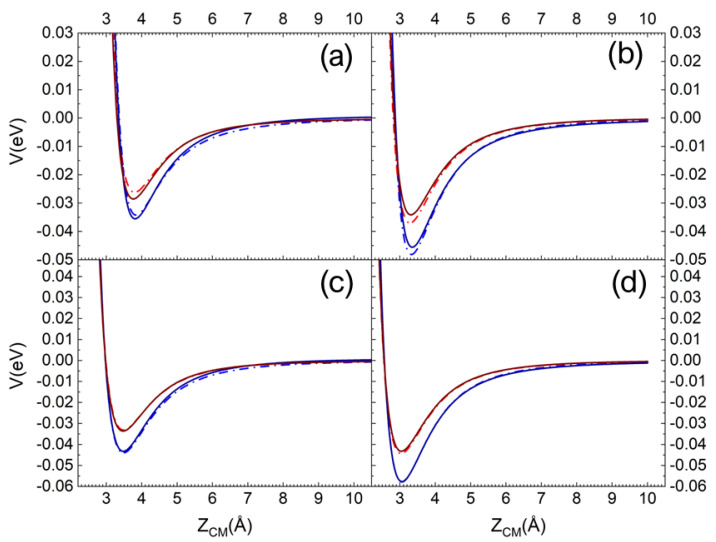
ILJ contribution (dash-dot line) and complete interaction potential (solid line) for N_2_ molecule interacting with molecular axis (**a**) perpendicular and (**b**) parallel to the surface plane on a Si surface atom. (**c**,**d**) the same but for N_2_ impinging on an O atom in the first sublayer. The red and blue lines are for the interaction on the cristobalite and quartz, respectively.

In Figure 1a,b, the pure long-range ILJ potential contribution and the complete (including the electrostatic component) V_N2-silica_ potential are reported as a function of the Z_CM_, the distance along the normal to the surface plane of the molecule centre of mass (CM). The different curves refer to N_2_, with the molecular axis aligned perpendicular and parallel to the surface plane, impinging on a Si atom (top layer). In Figure 1c,d, the same curves are reported for N_2_ impinging on an O atom (first sublayer). For an easy and immediate comparison in each panel, the curves for the interaction on the quartz (blue lines) and the cristobalite (red lines) are reported [29]. Looking at Figure 1, we can immediately appreciate that the N_2_ molecule interacts more strongly with quartz than with cristobalite. Furthermore, for a given polymorph, a stronger interaction manifests when the molecule interacts with the molecular axis parallel to the surface plane and the interaction directly involves an oxygen atom in the first sublayer (see Figure 1c,d).

Therefore, for the quartz, N_2_ in this molecular orientation interacts more strongly with its CM impinging on an O atom in the first sublayer than in the case of incidence on a Si atom.

It is also worth noting that the contribution of the Coulombian potential is very small in the case of interaction with the silicon atom, whereas for oxygen it is not even appreciable.

### 2.2. Potential Energy Surface Determination

**Figure 2:**
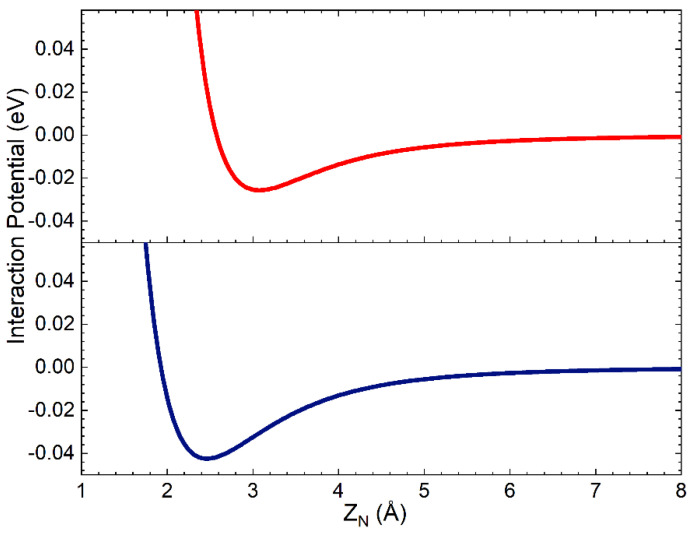
Interaction potential for the nitrogen atom interacting on the O atom in the first sublayer (red line) and on a Si atom in the second sublayer (blue line), in the assumed model crystal lattice, that involves, in the V_N-silica_ formulation, effective Z_N_ values larger than 4.0 Å.

The complete PES has the same functional form as that used in ref. [29], obtained by combining the term V_N-silica_, describing the interaction of a single N atom with the surface, with that for the interaction of N_2_ molecule with the same surface, V_N2-silica_, through a weight function depending on the interatomic distance r_N-N_. V_N-silica_ has been obtained by adding the interaction potential for short-range distances, resulting from the fitting procedure of the ab initio DFT calculations in ref. [30], to that for the long-range region modelled as an ILJ interaction potential (see previous Section and Section 3). Since the interaction of the N atom with silica clusters, cleaved by the crystallographic unit cell, has been proven to be a local component involving only the N impinging atom and Si (in the cluster) partners, we assumed the same short-range potential for both polymorphs. Furthermore, here we are interested in studying the interaction of molecules with the two polymorphs and, in the investigated collision energy range, we are expecting a minor influence of atomic potential on the collision dynamics. For all these reasons, the strong short-range potential for one N atom interacting with a Si atom on the surface is that used in ref. [29]. In Figure 2, the weak long-range interaction of N with an O atom in the first sublayer and with a Si atom in the second sublayer is reported as a function of the distance along the normal to the surface of the nitrogen atom, Z_N_. In the assumed reference frame, Z = 0 is on the top layer.

Looking at Figure 2, it appears that the N atom long-range interactions can be active and not negligible up to the second layer placed at 1.78 Å below the surface top layer.

**Figure 3:**
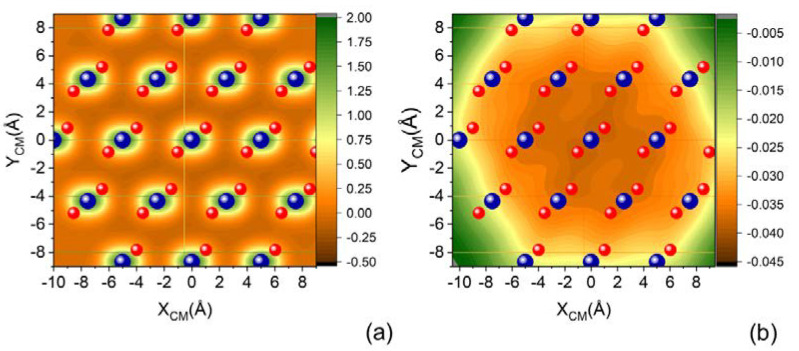
(**a**) XY projection of complete PES for N_2_ molecule impinging with molecular axis parallel to the XY plane of quartz surface for (**a**) Z_CM_ = 2.0 Å and (**b**) Z_CM_ = 3.5 Å.

Figure 3 shows two projections of a cut (parallel molecular approach to the surface) of the complete PES on the XY surface plane, corresponding to the molecular center of mass placed at two selected distances from the surface: Z_CM_ = 2 Å and Z_CM_ = 3.5 Å. We can appreciate that with increasing Z_CM_, the specificity of the sites corresponding to the Si and O atoms building the surface is lost and each SiO_2_ unit manifests almost as a single center of attraction.

### 2.3. The Dynamics of N_2_ Interacting with Quartz

**Figure 4:**
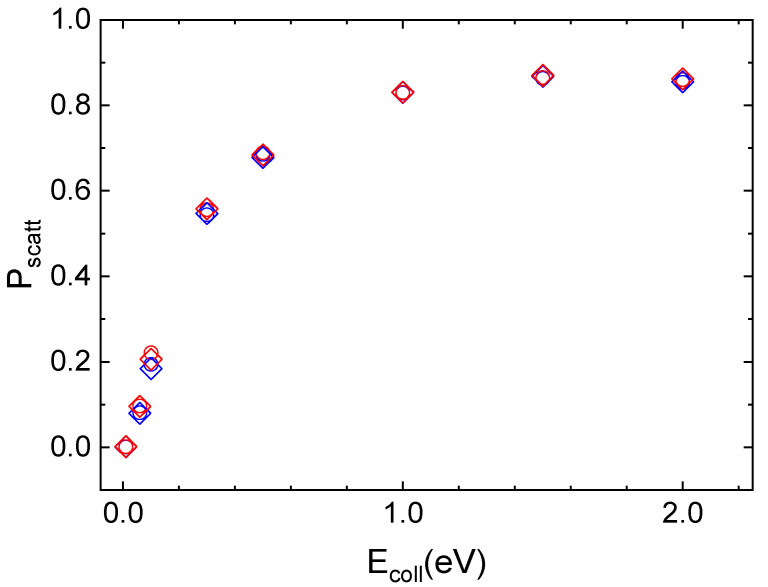
Inelastic probability for the roto-vibrational ground state of ortho (open circle) and para (open diamond) nitrogen molecules scattered by the quartz surface at T_S_ = 100 K (blue symbols) and T_S_ = 1000 K (red symbols).

The interaction of N_2_(v_i_,j_i_) molecules in the ground vibrational (v_i_ = 0) and rotational (j_i_ = 0 for *ortho* and j_i_ = 1 for *para* symmetries) states with the quartz surface leads to inelastic scattering, whose probability of occurring rises sharply as E_coll_ increases and then it assumes almost constant values at the highest collision energies, independently of v_i_ and j_i_ value. This trend can be seen in Figure 4, where the scattering probability for *ortho* and *para* nitrogen molecules, approaching along the normal to the surface plane, at the two considered surface temperatures, is reported.

For the three lowest collision energy values (E_coll_ ≤ 0.1 eV), the integration of most of the trajectories is stopped as the molecule reaches the edge of the crystal lattice by diffusing on the surface through single or multi-rebounds. These trajectories are those for which it is not possible to assign with certainty the final reaction channel, as they are neither completely adsorbed nor completely scattered off in the gaseous-phase. Instead, for E_coll_ ≥ 1.0 eV the possibility for molecular adsorption also opens up, so that the N_2_ molecule remains on the surface at the end of the trajectory. For N_2_/quartz interaction, unlike cristobalite, even at extremely low collisional energies, the second most likely process is the inelastic scattering, albeit with a low probability value. In the complete range of considered E_coll_ values, the initial vibrational state is preserved after the scattering from the surface.

**Figure 5:**
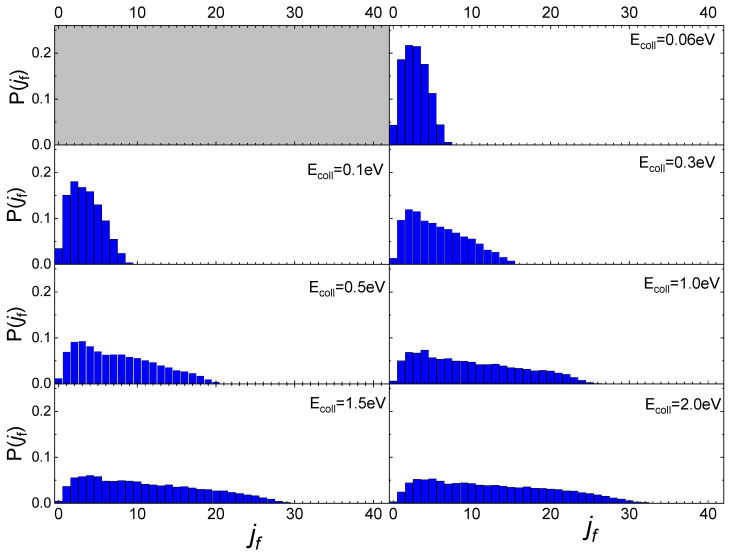
Final rotational distributions for N_2_(0,0) molecules scattered by quartz surface. T_S_ = 100 K.

In Figure 5 the final rotational distributions, obtained for the scattering of N_2_(0,0) molecules, are reported for T_S_ = 100 K. It appears that for E_coll_ = 0.06 eV the distribution has a sharp peak in the range between j_f_ = 1 and j_f_ = 4. The distributions widen and the peak height decreases as the collision energy increases. As for the lowest collisional energy value considered, E_coll_ = 0.01 eV, although the number of trajectories is too small to be statistically significant, it is observed that almost all trajectories end in j_f_ = 1.

**Figure 6:**
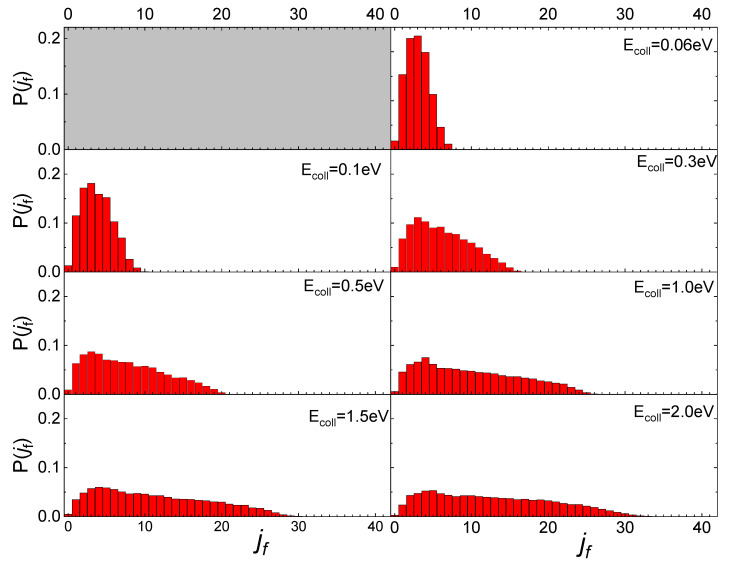
Final rotational distributions for N_2_(0,1) molecules scattered by quartz surface. T_S_ = 100 K.

Increasing the value of j_i_ to 1, the obtained final rotational distributions, shown in Figure 6, are very similar to those obtained for the lowest j_i_ value, only showing a slight propensity to increase by 1 the value of j_f_ corresponding to the maximum of the distribution in comparison with j_i_ = 0 case. This propensity is also confirmed at the lowest collision energy, despite the uncertainty about the probability values corresponding to this energy, due to the low trajectories number ending with the scattering.

These findings differ from those obtained for N_2_ molecules’ interaction with a graphite surface [13]. Firstly, the scattering of N_2_ molecules from a graphite surface takes significant values even at the lowest collision energy (E_coll_ = 0.01 eV) allowing us to define the final rotational distributions, while for the quartz, at that energy, the scattering events are very few. Secondly, the scattering of N_2_ from a quartz surface produces final rotational distributions with the peak in j_f_ = j_i_ + 2 for E_coll_ ≤ 0.3 eV, while for E_coll_ > 0.3 eV the j_f_ value corresponding to the distribution peak is independent of j_i_, appearing more closely related to the value of collision energy. Finally, no secondary maxima are observed in the region of high j_f_, bringing out the absence, for the interaction under study, of rotational rainbow scattering, already highlighted for the scattering of N_2_ from metal [31,32,33] and graphite [13] surfaces. 

Increasing the values of v_i_ to low-medium-lying values, v_i_ = 5 and v_i_ = 10, the interaction dynamics do not change as well as the rotational distribution features of scattered molecules, suggesting a direct correlation between the initial rotational state and the final rotational distributions of the scattered molecules as already observed for N_2_ molecules interacting on graphite [13].

**Figure 7:**
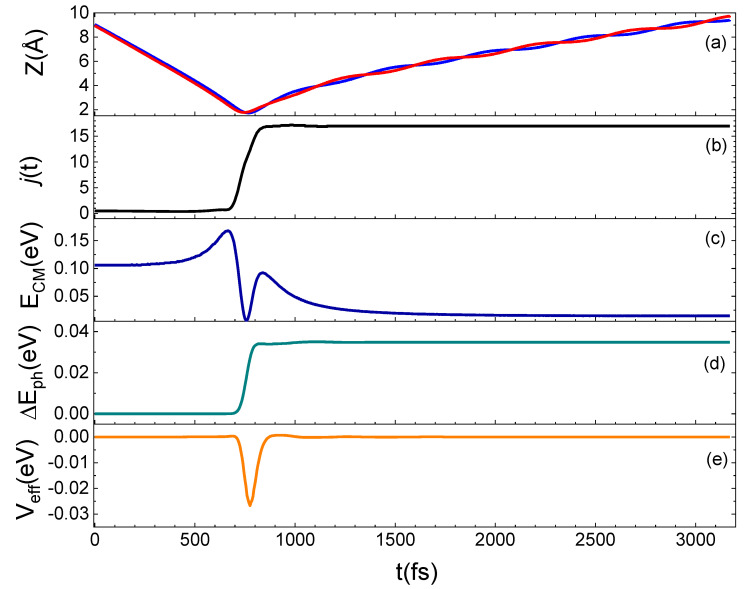
Trajectory for N_2_(0,0) at E_coll_ = 0.5 eV backscattered in the gas-phase as N_2_(0,17). (**a**): Dependence on the time t of the Z coordinate, for the two atoms (identified by red and blue lines) in the N_2_ molecule; (**b**): rotational state evolution along the trajectory; (**c**): variation of the CM translational energy; (**d**): energy exchanged with the surface phonons; (**e**): V_eff_ potential.

To better understand the microscopic mechanism underlying the interaction which determines the results presented above, we have examined in detail some prototypical trajectories. From this analysis, it emerges that the interaction occurs mainly through a direct mechanism, in the sense that the molecule is immediately backscattered in the gas-phase, although an indirect mechanism is also observed for some trajectories, mostly at the lowest collision energies or when the molecule impinges on the void space between pairs of Si atoms on the surface. Under such conditions, the molecule remains on the surface from a few tens to a few hundreds of femtoseconds before being backscattered. A relationship exists between the motion of the molecule after the interaction with the surface and the rotational state in which it is reflected in the gas-phase, as found for the N_2_ scattering from a graphite surface [13]. In fact, molecules leaving the surface moving with a *cartwheel*-like motion are scattered in high j_f_, while molecules moving with a *helicopter*-like motion have a low j_f_. Trajectories are also observed for which the internal motion of the molecule changes as an effect of the interaction with the surface so that N_2_ incident with a *helicopter*-like motion leaves the surface moving with a *cartwheel*-like motion. In Figure 7, an example of this trajectory kind is shown. Here, we report the time evolution along a typical trajectory of Z (the distance along the normal direction to the surface plane of both N atoms in the molecule), of the rotational number j, of the translational kinetic energy of the molecule center of mass (E_CM_), of the energy exchanged with the surface phonons (ΔE_ph_) and of the effective potential (V_eff_), describing the perturbation of impinging molecule to the motion of surface atoms. The N_2_ molecule impinging in the lowest ground-state is inelastically reflected in the gas-phase in j_f_ = 17.

The translation–rotation coupling is effective when the molecule comes close to the surface. For N_2_ molecules interacting with the quartz surface, such coupling has also been highlighted in ref. [19] by using a different model potential. Therefore, we can claim that the translation–rotation energy exchange is strictly related to the long-range interactions, governing the scattering of molecules weakly interacting with surfaces, through physisorption states.

### 2.4. Effect of Surface Polymorph: N_2_/Cristobalite and N_2_/Quartz

**Figure 8:**
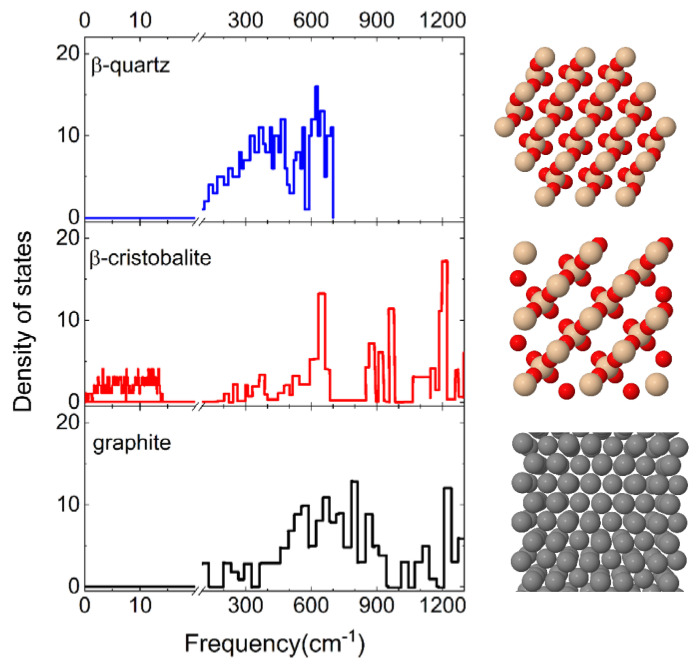
(**left**) Phonon frequency distributions of the β-quartz [34], β-cristobalite surfaces [35]. The distribution for the graphite surface [36] is also reported for comparison, being useful in the discussion. A break is inserted on the frequency axis between 20 cm^−1^ and 100 cm^−1^. (**right**) Top view of crystal structures of the corresponding material.

The effect of the surface polymorph on N_2_ molecule interaction is highlighted by comparing the results presented in the previous paragraph with those obtained for cristobalite in ref. [29]. Firstly, it is found that when N_2_ molecules impinge on a cristobalite silica surface, the dominant process, regardless of the initial internal state and of the collision energy, is the molecular adsorption process, whereas the inelastic scattering has an extremely low probability of occurring. Instead, as shown in the previous Section, when N_2_ molecules collide with a quartz silica surface the inelastic scattering plays a relevant role, becoming the dominant process at the intermediate and high values of probed E_coll_. Different findings resulted from the investigation of N_2_ interaction on a graphite surface [13] in which molecular adsorption occurs only at the lowest collision energies. Therefore, it appears that both the chemical composition and the crystallographic structure of the surface material have a great influence on the dynamics of a given interacting species. The only difference between quartz and cristobalite is the way of binding the tetrahedral SiO_4_ unit in the 3D structure of the surface, while both have a different composition with respect to graphite. Therefore, while the cristobalite exhibits a free region on the surface in which molecules can be trapped, the quartz, due to the displacement of the SiO_4_ tetrahedra within different layers, has a more compact structure, thus limiting the chances for the molecule to precipitate below the first layer (see Figure 8).

These structural and chemical differences lead, as a consequence, to a different distribution of the phonon states, explicitly considered in our calculations, and reported in Figure 8. It emerges that cristobalite exhibits a band at extremely low frequencies (<20 cm^−1^) that is missing in the spectra of quartz and graphite. Indeed, the first significant band in the graphite phonon state density is centered around the 20 THz (~667 cm^−1^): this spectrum region corresponds to the maximum and intermediate region of the quartz and cristobalite spectrum, respectively. Therefore, the silica surfaces exhibit a greater density of states at low frequencies than graphite. This circumstance is of primary importance for gas–surface heterogeneous reactions, meaning that this frequency region corresponds to the phonons associated with the surface atoms. Thus, the different behavior of quartz is attributable to the different structures of the phonon spectrum. In fact, N_2_ molecules interacting with cristobalite undergo a strong coupling with the surface contrary to what occurs on quartz where the coupling is much lower and the molecules are more efficiently reflected.

**Figure 9:**
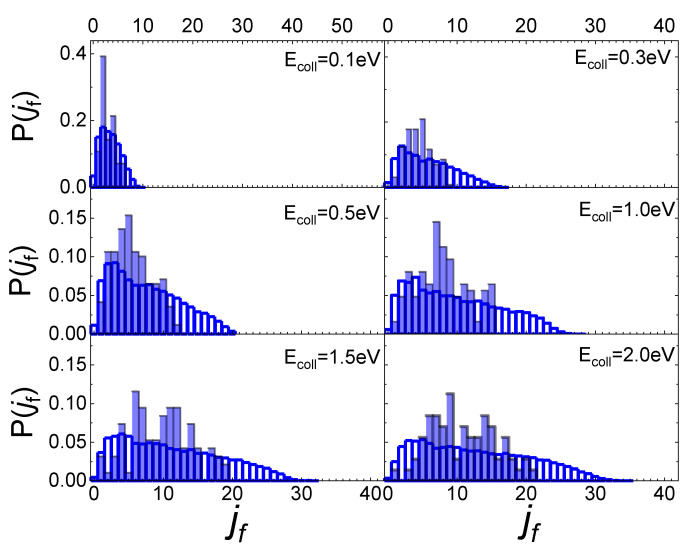

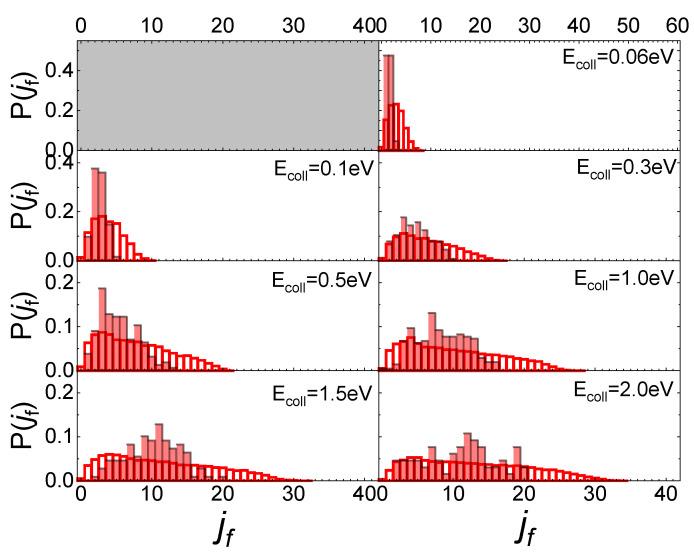
Final rotational distributions for ortho (**upper panel**) and para (**lower panel**) N_2_ molecules inelastically scattered by quartz (in foreground) compared with those obtained in ref. [29] for cristobalite (in transparency) as a function of the collision E_coll_ of impinging molecules.

The influence of the phonon dynamics manifests not only in the occurring surface processes but also in the rotational distributions of the N_2_ molecules inelastically scattered by the two silica polymorphs considered here. In Figure 9, the determined distributions for quartz are reported in comparison with those recently obtained for cristobalite [29]. As mentioned in the previous paragraph, looking at Figure 9, it appears that at low E_coll_ the rotational distributions of molecules scattered by quartz exhibit relative peak heights with a maximum for j_f_ < 5 and then gradually decline by extending towards higher j_f_ values as E_coll_ increases. On the contrary, those for cristobalite have a more symmetrical trend around a maximum, that moves to higher values of j_f_ as E_coll_ increases. The distributions here determined for N_2_(v_i_, j_i_) scattered from quartz are very similar to those obtained in ref. [13] for N_2_(v_i_, j_i_) molecules scattered from graphite, although in the case of quartz the secondary peaks at high j_f_ values, ascribed to rotational alignment and rotational rainbow already discussed in refs. [12,13], are missing.

Despite the emergence of differences, both in the probabilities for the various surface processes and the final rotational distributions, the mechanism underlying collision events is the same for the three surfaces, essentially based on the exchange between rotational and translational molecular internal degrees of freedom, as N_2_(v_i_, j_i_) approaches the surface. How efficient this exchange is at promoting molecular adsorption or inelastic scattering depends on the topology and energy put into play by the phonons participating in the interaction. The effect of the polymorph on the rotational inelasticity becomes evident in the final rotation distributions that are generally more specific to cristobalite, whereas for quartz they extend to higher values of j_f_.

### 2.5. Surface Temperature Effect

#### 2.5.1. N_2_/Quartz

Looking at Figure 4 in Section 2.3, the interaction dynamics of N_2_ molecules in the two roto-vibrational ground states appears independent of surface temperature. A slight surface temperature effect for *ortho* and *para* N_2_ molecules in the ground vibrational state is evident only for E_coll_ ≤ 0.1 eV and manifests with an increase in the population probabilities for the final rotational states placed to the right of the main peak, which roughly corresponds to the same value of j_f_ for the two surface temperature values. The trend of rotational distributions is preserved and no temperature effect is observed by increasing the initial vibrational state.

**Figure 10:**
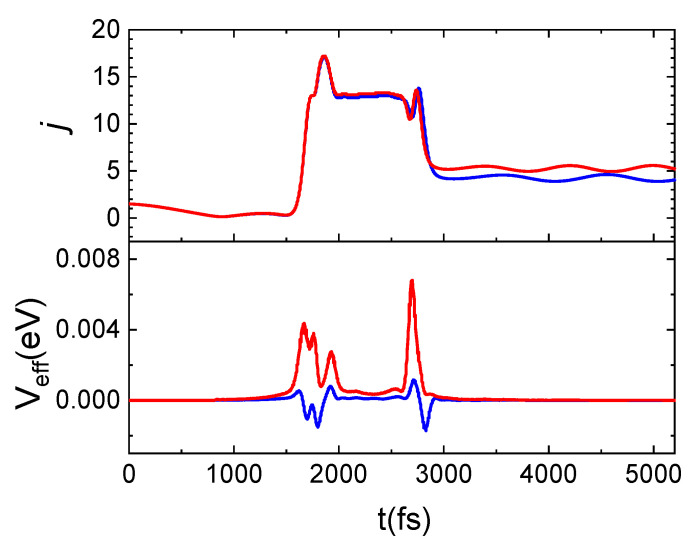
Time evolution for j and V_eff_ (see text) for the same trajectory propagated at T_S_ = 100 K (blue lines) and T_S_ = 1000 K (red lines) for N_2_(0,1) and E_coll_ = 0.06 eV.

A careful examination of the trajectories enables us to ascribe the increment in the population of higher rotational states to the energy contribution of surface phonons, depending on the T_S_ value. Indeed, the same trajectory integrated at the two considered T_S_ values exhibits the main difference in the potential term, V_eff_, as can be inferred by looking at Figure 10. Here, for the same trajectory, propagated at the two considered surface temperatures, the evolution time of the rotational number and V_eff_ are reported. N_2_(0,1) is scattered in N_2_(0,4) for T_S_ = 100 K and in N_2_(0,5) for T_S_ = 1000 K. Again, from Figure 10, it emerges that the *j* increment is attributable to the positive and larger contribution of V_eff_ with respect to what happens at the lowest temperature for which this term is mainly negative. In particular, the net increment/decrement of V_eff_ occurring around 1800 fs and 2800 fs correspond to the instant in which the molecule comes close and leaves the surface. Instead, in the range 1800–2800 fs, V_eff_ remains almost constant and the difference in the values assumed by this latter at the two temperatures is almost imperceptible. This trend is due to the fact that in this time-lapse the molecule comes close to the surface and changes its motion, beginning to rotate. Then, the molecule moves away from the surface by moving with a *cartwheel*-like motion, different from the one with which it had impacted.

#### 2.5.2. N_2_/Cristobalite

**Figure 11:**
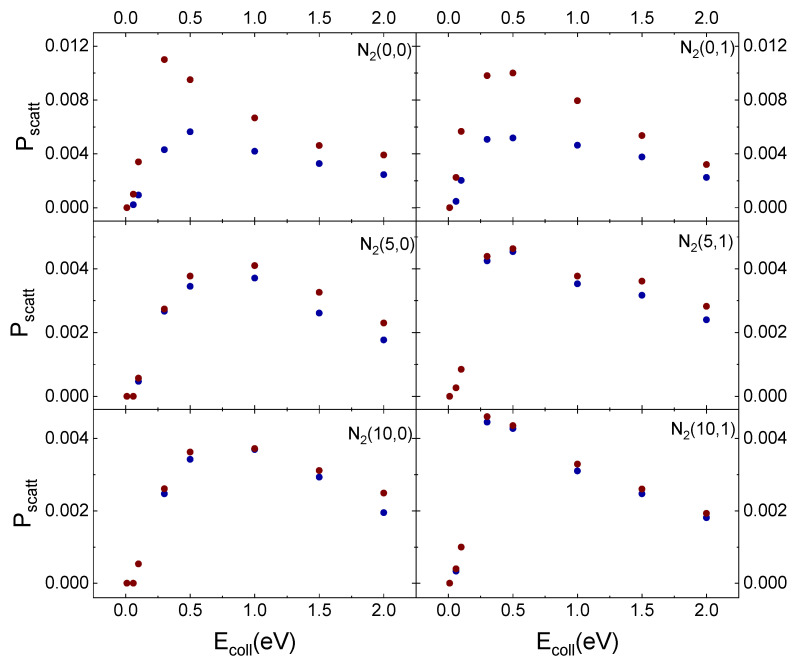
Probability (P_scatt_) for inelastic scattering of N_2_(v_i_,0) and N_2_(v_i_,1) from cristobalite for T_S_ = 100 K (blue solid dots) [29] and T_S_ = 1000 K (red solid dots) as a function of the collision energy.

In Figure 11, the results obtained for inelastic scattering of *ortho* and *para* N_2_ molecules in the different initial vibrational states from the cristobalite surface, taken at T_S_ = 1000 K, as a function of E_coll_ are displayed in comparison with those obtained in ref. [29] for T_S_ = 100 K. 

The temperature effect is evident in Figure 11 for ortho and para nitrogen in the ground vibrational state. However, even at the highest surface temperature, the difference in the scattering probability for the two species is perceptible only for the lowest considered collision energy values. Instead, increasing v_i_ values, this effect is lost. The surface temperature effect is observed only for v_i_ = 0 because molecules, although mostly following the same trajectories, at the lowest T_S_ values undergo a more efficient interaction with the surface. Therefore, some trajectories ending with scattering at the highest T_S_ value, at T_S_ = 100 K, evolve by molecular diffusion on the surface, terminating when the molecule reaches the lattice edges or adsorbs. Furthermore, since the interaction is stronger at the lowest temperature, the molecules show a greater energy exchange which causes a redistribution of energy at the play, leading to a decrease in the CM translational energy and an increase in the molecule rotational energy content. These findings highlight once again the role of translation–rotation coupling.

On the other hand, a nitrogen molecule initially in v_i_ = 5 or v_i_ = 10 holds an energy content sufficient to counteract the interaction with the surface, even at the lowest temperature. Accordingly, the molecule is scattered inelastically with the same probability as that at the highest temperature. The higher scattering probability for v_i_ = 0, for both T_S_ values, can be directly related to the corresponding lower energy content responsible for molecule trapping in the physisorption well from which, through rebounds on the repulsive potential wall, it is re-emitted. Instead, increasing v_i_ to 5 or 10 the highest available energy determines that the molecule does not remain trapped in the well and can come much closer to the surface. It follows that a large number of trajectories end with molecular adsorption, perhaps also due to the increased effect of the electrostatic potential.

## 3. Methods

### 3.1. Computational Setup

The computational setup, used to describe the collisions of N atoms/N_2_ molecules with silica surfaces, is based on a semiclassical collisional approach [23]. The method has already been used in the past [12,13,30,34,37,38,39] to study molecule formation and inelastic scattering on surfaces. More recently, it has been adopted to describe the collision dynamics of N and N_2_ with a silica (cristobalite) surface [29]. However, a brief outline of the most important and specific features of the method is here provided.

The dynamics of gas-phase particles is followed by solving the relevant 3D Hamilton’s equations of motion self-consistently with the dynamics of the lattice phonons. 

The complete Hamiltonian for the two gas-phase atoms interacting with the surface is given by:

(1) $$H=\frac{1}{2}\sum_{i}\frac{P_{i}^{2}}{m_{i}}+V\left(r\right)+{\Delta E}_{\mathrm{ph}}+V_{\mathrm{eff}}\left(t,{\;T}_{S}\right)\;\;\;\;\;i=1,2$$

where Pi is the momentum of atom i having mass mi, V(r) is the interatomic potential of the gas-phase species, ΔE_ph_ is the energy exchanged with the surface phonons. V_eff_(t, T_S_) is the effective potential of the mean field type which depends upon the interaction time and the surface temperature. According to the Ehrenfest theorem, V_eff_ is obtained as the expectation value of the interaction potential between the atoms in the gas-phase and the surface (V_int_) on the phonon wave function, that is:

(2) $$V_{\mathrm{eff}}\left(t,{\;T}_{S}\right)=\langle \Psi \left(t,T_{S}\right){|V}_{\mathrm{int}}\left(R\right)|\Psi \left(t,T_{S}\right)\rangle$$

R being the two-body distance between each gas-phase atom and the lattice atoms. Ψ (t,T_S_) is the total wave function of the phonon modes given as a distribution of quantum states at a fixed surface temperature T_S_:

(3) $$\Psi \left(t_{0},T_{S}\right)=\prod_{k=1}^{M}|n_{k}^{0}\rangle$$

The time evolution of the phonon wave function is given by analytically solving the time-dependent Schrödinger equations of motion under the harmonic oscillator approximation, that is assuming that a set of *M* = 3*N_at_* − 6(*N_at_* is the number of atoms in the 3D slab) independent harmonic oscillators are perturbed by a linear (and quadratic) force exerted between the atom/molecule approaching the surface from the gas-phase and the solid substrate. Thus, by expanding the interaction potential in the normal mode coordinates up to the linear term, the following expression is obtained for the effective potential of Equation (2):

(4) $$V_{\mathrm{eff}}\left(t,{\;T}_{S}\right)=V_{0}+\sum_{k}V_{K}^{\left(1\right)}\eta_{k}\;\left(t\right)$$

where V_0_ is the ‘‘static’’ interaction potential between the atoms in the gas-phase and the lattice atoms in their equilibrium positions. $V_{K}^{\left(1\right)}=\frac{\partial V_{\mathrm{int}}}{\partial Q_{K}}\;{|}_{eq}$ is the linear driving force exerted on each k-th phonon mode Q_k_. η_k_(t) are the ‘‘phonon excitation strengths’’ given in terms of the Fourier components I_i,k_(t) of the external force:

(5) $$\eta_{k}\left(t\right)=-\int d\;t^{\prime }{\left(\hbar \omega_{k}\right)}^{-1}\frac{\partial }{\partial \rho_{k}}({\Delta E}_{k}^{+}+{\Delta E}_{k}^{-})[I_{c,k}\left(\;t^{\prime }\right)\cos\left(\Theta_{k}\left(\;t^{\prime },\omega_{k}\right)\right)+I_{s,k}\left(\;t^{\prime }\right)\sin\left(\Theta_{k}\left(\;t^{\prime },\omega_{k}\right)\right)$$

(6) $$I_{c,k}=\int_{-\infty }^{+\infty }{\mathrm{dtV}}_{k}^{\left(1\right)}\left(R\left(t\right)\right)\cos(\omega_{k}t)$$

With $\Theta_{k}\left(t,\omega_{k}\right)\approx \omega_{k}t,$ $\omega_{k}$ being the frequency of the *k*-th phonon mode. ${\Delta E}_{k}^{\pm }\left(\omega_{k},{\;\rho }_{k}^{\pm };{\;T}_{S}\right)$ is the energy exchanged between the chemical particles in the gas-phase and the solid substrate due to the phonon creation (${\Delta E}_{k}^{+}$) and phonon annihilation (${\Delta E}_{k}^{-}$) processes in the *k*-th phonon mode.

The energy exchanged with the phonons can be obtained from the transition probabilities $P_{n_{k}^{0}\;\to n_{k}}$ for the excitation/de-excitation phonon processes and is given by:

(7) $${\Delta E}_{\mathrm{ph}}=\sum_{k}\sum_{n_{k}}\sum_{n_{k}^{0}}p_{n_{k}^{0}}\left(E_{n_{k}}-E_{n_{k}^{0}}\right)P_{n_{k}^{0}\;\to n_{k}}=\sum_{k}({\Delta E}_{k}^{+}+{\Delta E}_{k\;}^{-})$$

where $\;\left(E_{n_{k}}-E_{n_{k}^{0}}\right)$ is the energy exchanged in the transition $n_{k}\leftarrow n_{k}^{0}\;$ between the quantum state $n_{k}^{0}$ and $n_{k}$ of the *k*-th phonon mode, and $p_{n_{k}^{0}}\;$ is the Boltzmann distribution of the phonon energies.

Finally, the dynamics of the nuclear motions of each gas-phase atom interacting with the surface is obtained by integrating the classical Hamiltonian equations of motion:

(8) $${\overset{\cdot }{R}}_{1}=\frac{\partial H}{\partial P_{i}}\;\;\;\;{\overset{\cdot }{P}}_{1}=\frac{\partial H}{\partial R_{i}}$$

Then, the complete dynamical simulation is worked out through three main steps:

(1) A 3D slab of considered material is built up from crystallographic data or from ab initio calculations and the corresponding phonon dynamics is determined.
(2) An accurate interaction potential for the [gas-phase species/surface] system is obtained as sum of pair-wise interaction starting from ab initio calculations.
(3) The dynamics is carried out by self-consistently solving Hamilton’s equations of motion of the two interacting atoms [Equation (8)] and the dynamics of the phonons, i.e., by computing the phonon excitation strengths given in Equation (7) at each time step of the classical trajectory.

The surface models, adopted here for the two silica polymorphs, are those of refs. [34,35] for quartz and cristobalite, respectively. The phonons dynamics for these 3D surface models has been determined there.

Recently, the need to include a proper formulation of the long-range attraction in the gas–surface interaction has been demonstrated, since it is promoting the formation of the precursor state that controls all basic elementary processes occurring at the gas–surface interphase [10,12,13,14,24,25]. Therefore, in the present study, we have evaluated the long-range interaction potentials, represented by the improved Lennard–Jones ILJ model [26], for N/N_2_ impinging on quartz, inserted in the new PES formulation (see next Section 3.2), whilst the PES for the interaction of N/N_2_ with cristobalite was lately determined in ref. [29].

The adopted method treats the molecular translational motion classically, whereas the rotational and vibrational degrees of freedom of scattered molecules (the latter, represented as Morse oscillators [40]) are analyzed in terms of the action-angle variables by using the semiclassical quantization rules [41]. Accordingly, at each time, the internal energy of the interacting molecule is partitioned into vibrational and rotational contributions by using equations that include rotational angular momentum and vibrational spectroscopic constants. We are unable to predict some features due to quantum effects (tunnelling) and selection rules (forbidden transitions between the different roto-vibrational levels); however, at least in the choice of the initial molecular states, we distinguished between *ortho* and *para* nitrogen.

### 3.2. PES Formulation Details

As for N,N_2_/cristobalite [29], the multidimensional PES describing the interaction N,N_2_/quartz has been obtained by combining the interaction potential of the molecule, $V_{N_{2}\text{-}\mathrm{quartz}}$, with that of the atom, V_N-quartz_, through a weight function, f_sw_(r_N−N_), that permits a smooth transition from N_2_–quartz to N–quartz interaction potential as r_N−N_, the intramolecular distance, increases. In particular, f_sw_(r_N−N_) provides quick switching between 1 and 0, preserving the values of $V_{N_{2}\text{-}\mathrm{quartz}}$ and V_N-quartz_ for r_N−N_ lower and higher, respectively, of the intramolecular distance (r_diss_ = 3.5 Å) considered critical for N_2_ molecule dissociation. In the range of distances where the switch occurs, the potential is obtained as a combination of both components, weighted according to the f_sw_(r_N−N_) value. To avoid discontinuity points in the transition from N_2_–quartz to N–quartz interaction representation, a continuity check of the obtained potential function has been carried out.

The adopted PES is represented as a sum of pair-wise interactions between the free atom or each effective atom (i.e., in a state different from the isolated one) in the molecule and each effective atom of the crystal lattice. In particular, it is formulated as:

(9) $$V\left(r_{N-N}\;,\;R\right)=\sum_{i=1}^{N_{at}}\sum_{k=1}^{2}V_{N_{2}\text{-}\mathrm{silica}}\left(R_{\mathrm{ik}}\right){*f}_{\mathrm{sw}}\left(r_{N-N}\right)+\sum_{i=1}^{N_{at}}V_{N\text{-}\mathrm{silica}}\left(R_{i}\right)*\left(1-f_{\mathrm{sw}}\left(r_{N-N}\right)\right)$$

where $V\left(r_{N-N}\;,\;R\right)$ is the total interaction potential. In the first term, on the right side of Equation (1), the variable R is the distance between the i-th atom in the lattice and the k-th N atom in the molecule, while, in the second term, it represents the distance between the i-th atom in the lattice and the gaseous N atom. The analytical expression of f_sw_($r_{N-N}$) is the same used in ref. [13]. 

According to the ILJ formulation, each (atom, effective atom)−(effective atom) pair interaction contribution, between N or N(in N_2_) and each atom in the lattice, is obtained as:

(10) $$V_{\mathrm{ILJ}}\left(R\right)=\varepsilon \left[\frac{m}{n\left(R\right)-m}{\left(\frac{R_{m}}{R}\right)}^{n\left(R\right)}-\frac{n\left(R\right)}{n\left(R\right)-m}{\left(\frac{R_{m}}{R}\right)}^{m}\right]$$

with

(11) $$n\left(R\right)=\beta +4{\left(\frac{R}{R_{m}}\right)}^{2}$$

The first term of Equation (10) describes the size repulsion, whereas the second one the dispersion attraction associated with each interacting pair. The parameters ε and R_m_, which represent the potential well depth and its location, respectively, associated with each considered pair, define the strength of both terms in Equation (10). An extended discussion on their choice is presented in the next Section 3.3. Moreover, *β* is an additional parameter depending on the “hardness” of the two partners. For the neutral–neutral interactions, as the present ones, *β* has been fixed to 7.5 and m = 6 has been used. For each interacting pair, the ILJ function provides an asymptotic dispersion attraction contribution, related to a partial C_6_ coefficient defined as C_6_ = ε∙R_m_^6^. The combination of all partial C_6_ coefficients determines the value of the global atom/molecule–surface attraction coefficient C_3_ [42] which controls capture efficiency and formation of the precursor state of gas–surface elementary processes.

### 3.3. Polarizability and Potential Parameters

Electronic polarizability is considered the key property controlling strength and range of the fundamental components of non-covalent long-range interactions. Basic correlation formulas between polarizability and potential parameters, corresponding to the potential well ε the minimum position R_m_ and the long-range attraction coefficient C_6_, have been proposed some years ago (see ref. [43] and references therein) and exploited here. Since the polarizability distribution in the SiO_2_ unit was not known, estimated effective-atomic components of polarizability have been initially assumed. In more detail, the starting values of the ILJ potential parameters, namely ε, R_m_ and related C_6_ attraction coefficient (see the previous Section 3.2), were obtained by partitioning the polarizability of SiO_2_ into the two contributions due to O and Si atoms in the monomer unit. Then, the polarizability contributions were modified, leaving that of the O atom unchanged and decreasing that of Si atom more uncertain due to the polar bond. We obtained a sequence of ε values in agreement with those reported in the literature, where it is found that the most important centers of interaction are located on the O atoms [21,22,44]. Therefore, the value of ε for the interaction with each O atom must be greater than that with the Si atom. This assumption is also confirmed in ref. [45], where the effective polarizability of Si in SiO_2_ is smaller than that of O due to the polarity of the bonds.

Accordingly, the used final values for the N-SiO_2_ interaction were selected by comparing the value of the so-derived dispersion coefficient C_6_ with that describing the attraction of the single monomer unit. The adopted ε and R_m_ parameters, equal to 7.30 meV and 3.67 Å for N-O (in SiO_2_) and 4.86 meV and 3.67 Å for N-Si (in SiO_2_), give partial C_6_, respectively equal to 1.78 × 10^4^ meVÅ^6^ and 1.18 × 10^4^ meVÅ^6^. The global (N-SiO_2_) C_6_ value of 4.74 × 10^4^ meVÅ^6^ for the single monomer unit is in good agreement with the value of 4.49 × 10^4^ meVÅ^6^, that results by combining the C_6_ for the single coefficient contributions reported in ref. [20], equal to 0.59 × 10^4^ meV Å^6^ for the N-Si interaction (Si in SiO_2_) and 1.95 × 10^4^ meV Å^6^ for the N-O interaction (O in SiO_2_), respectively. 

The same method has been adopted to obtain the parameters for the interaction of N_2_ molecule with the SiO_2_ unit. A partial value of C_6_ = 1.48 × 10^4^ meV Å^6^, with R_m_ = 3.62 Å and ε = 6.59 meV, is estimated by us for the interaction of N (in N_2_)-O (in SiO_2_). Previously [20], for N_2_-O (in SiO_2_) it was found a global value of C_6_ = 2.97 × 10^4^ meV Å^6^ with R_m_ = 3.79 Å and ε = 10.02 meV (if, as indicated in ref. [20], R_m_ = 3.66 Å is assumed, ε = 12.35 meV would be obtained). Similarly, for N (in N_2_)-Si (in SiO_2_) we obtain partial C_6_ = 0.99 × 10^4^ meV Å^6^ with R_m_ = 3.62 Å and ε = 4.38 meV. Therefore, the global C_6_ for each N_2_-SiO_2_(unit) pair proposed by us amounts to 7.90 × 10^4^ meV Å^6^

However, results in the literature do not show uniqueness in the reciprocal relationship of the interaction components between N and the Si/O atoms in the lattice crystal. We determined the parameters by assuming a ε value for the interaction with the oxygen atoms greater than that due to the interaction with silicon atoms, but not as drastically as reported in ref. [20]. In addition, for the global interaction of the SiO_2_ monomer unit with each N atom, a value of C_6_ ≈ 6.00 × 10^4^ meV Å^6^ is reported in ref. [19], obtained by considering a value of ε for the interaction of N with O atoms (in SiO_2_) equal to about half of that for the interaction with Si atom (in SiO_2_). However, in this latter work, the assumed potential is a modified Morse function that works well to describe van der Waals interactions in the region of minima but not at long-range distances. In fact, the MSV (Morse–Spline–van der Waals) model [46,47] was used for a long time to partially overcome these limits, obviated with the formulation of the ILJ potential [26]. 

Furthermore, the Lorentz and Berthelot combination rules, often exploited to obtain the parameters for pair interactions should be used with great caution as they are effective only for very similar pairs of partners interacting through pure van der Waals forces. The hierarchy of values of ε for the interactions with the two elements in the crystal structure is respected in ref. [48], although perhaps too unbalanced in the interaction with the oxygen atoms, and this leads to a partial C_6_ value of 7.75 × 10^4^ meV Å^6^ for N-O (in SiO_2_). Instead, in ref. [49], the hierarchy for the values of ε is not respected and the parameters of the LJ potential have been defined using the Lorentz and Berthelot combination rules. In this case, the C_6_ coefficient for the N atom interacting with the SiO_2_ unit amounts to 3.41 ×10^4^ meV Å^6^. 

It should also be noted that when the traditional LJ potential is used for an interacting pair, if the depths of the physisorption wells are described correctly, the obtained C_6_ is double the correct value. Instead, if the long-range dispersion coefficient is the correct one, the value of the depth of the potential well must necessarily be either underestimated or moved to shorter distances from the surface. This inconsistency results from the fact that the relation for C_6_ = 2∙ ε ∙R_m_^6^, adopted by the classical LJ (12,6) model, is not correct, while in the ILJ model the proper relation is given above. A confirmation of these claims comes from the fact that in most of the considered cases the C_6_ obtained using the parameters of LJ potential are higher than the correct ones determined by us and those reported in ref. [20].

### 3.4. Molecular Dynamics Simulations

MD simulations, as described in Section 3.1, involve the integration of the Hamiltonian equations (Equation (8)) describing the interaction for fixed initial conditions and for each interacting species. For each considered set of initial roto-vibrational states and collision energy, we propagate and examine 30,000 trajectories. The molecule CM impinge with polar angle θ = 0° (defining the selected normal approach) and azimuthal angle (φ) of the molecular axis randomly determined for each trajectory. The surface temperature (T_S_) is taken at two different values T_S_ = 100 K and T_S_ = 1000 K. The initial coordinates of both atoms in the molecule are chosen randomly in an aiming area on the surface. This area is large enough and at the same time such as to prevent the edge effects during the trajectory propagation. The integration step is 0.20 fs and the accuracy required in the integration procedure is 10^−8^. In addition to the roto-vibrational ground state, we considered the medium excited vibrational states v_i_ = 5 and v_i_ = 10 for the two ground rotational states j_i_ = 0, 1. The CM collision energy (E_coll_) has been spanned on a wide range of values, in the range [0.01–2.0] eV.

We can describe and follow the different elementary surface processes occurring when the N_2_ molecule impinges on the surface (i.e., molecular scattering, molecular adsorption, dissociative adsorption), assumed as the PES reactive. The impact of a molecule on a surface can give rise to the adsorption of both atoms, to the desorption of just one atom with the other adsorbed on the surface, or to the desorption of both atoms.

## 4. Conclusions

The dynamics of collision between nitrogen molecules and a quartz surface has been studied by MD simulations adopting a new determined PES accounting for a suitable treatment for long-range interactions. Impinging N_2_ molecules are mainly inelastically scattered in the same vibrational state and in rotational states producing distributions exhibiting sharp peaks for j_f_ < 5 at low collision energies. Instead, the peaks attenuate themselves and the distributions widen to higher values of j_f_, increasing E_coll_. The comparison of these results with those reported in ref. [29] for another silica (cristobalite) surface highlights the role of surface polymorph in molecular scattering. Indeed, for silica (cristobalite), the leading surface process is molecular adsorption, whereas for silica (quartz) the inelastic scattering plays a relevant role, becoming the dominant process at intermediate and high collision energy. This finding represents an effect of the different surface’s atom packing.

The effect of surface temperature, evident only for silica (cristobalite) and low collision energies, is closely connected with the dynamics of surface phonons. In fact, the spectrum of the density of phonon frequencies only in the case of cristobalite has a band at very low frequencies, those precisely attributable to surface atoms.

For all considered surfaces, the coupling between the translational and rotational molecular internal degrees of freedom, which manifests itself by producing distributions pumped on higher levels than the initial one, plays a primary role in the scattering process.

## Data Availability

Data of this study are available upon request from the authors.
